# Expert consensus guidelines for community pharmacists in the management of diabetic peripheral neuropathy with a combination of neurotropic B vitamins

**DOI:** 10.1080/20523211.2024.2306866

**Published:** 2024-02-07

**Authors:** Thanompong Sathienluckana, Sirinoot Palapinyo, Kitiyot Yotsombut, Ekgaluck Wanothayaroj, Pasiri Sithinamsuwan, Naeti Suksomboon

**Affiliations:** a Faculty of Pharmacy, Siam University, Bangkok, Thailand; b Faculty of Pharmaceutical Sciences, Chulalongkorn University, Bangkok, Thailand; c Diabetes and Thyroid Center, Theptarin Hospital, Bangkok, Thailand; d Faculty of Medicine, Phramongkutklao Hospital, Bangkok, Thailand; e Faculty of Pharmacy, Mahidol University, Bangkok, Thailand

**Keywords:** Diabetic peripheral neuropathy (DPN), neuropathy, neurotropic B vitamins, pharmacist

## Abstract

This consensus guidance is for community pharmacists in diabetic peripheral neuropathy (DPN) management with a combination of neurotropic B vitamins. A multidisciplinary team including endocrinology, neurology, and pharmacy from Thailand discussed and aligned the practical scheme of DPN management in the community pharmacy setting, using the literature review and having face-to-face meeting. Five major statements have been endorsed as consensus recommendations for DPN care with strong acknowledgment. The aims of DPN management included reducing symptoms and the risk of complications, minimising adverse reactions from treatment regimens, and improving patients’ knowledge and adherence to the treatment strategies. An initial screening process using a 7 items interview of Douleur Neuropathique 4 (DN4) questionnaire should be implemented to identify patients at risk of developing DPN. Subsequently, pharmacologic, and non-pharmacologic treatment should be employed based on patient-centered care. An interesting approach is combination of neurotropic B vitamins, which may be used as monotherapy or combination therapy to control DPN symptoms. The combined therapy potentially exhibits a synergistic effect and improves patient adherence. The consensus would be further considered in context of harmonisation of routine practice and country requirements.

## 1. Introduction

Peripheral neuropathy (PN) defines damaged peripheral nerves resulting from many diseases. Diabetes is one of the common causes of peripheral neuropathy. Patients with diabetic peripheral neuropathy (DPN) may exhibit both positive and negative symptoms, where positive symptoms are painful or over-sensitized sensations, such as burning, electrical shock-like, and cold allodynia. While negative symptoms refer to a loss of regular sensory experiences like numbness and hypoesthesia (Inoue et al., 2016). The absence of protective sensation in patients with negative symptoms increases their susceptibility to foot ulceration. DPN can lead to severe conditions like ulcers, gangrene, and amputation, regardless of symptom manifestations. Some research reported that DPN accounts for 50–70% of non-traumatic amputations causing a massive loss of economy and productivity (Adler et al., 1999). Community pharmacists can play a crucial role in empowering patients to be aware of disease, early screenings, and treatment to prevent disease progression because patients can simply access community pharmacist’s counselling non-pharmacological and pharmacological approaches (Bodman & Varacallo, 2022). Neurotropic B vitamins (B1, B6, and B12) are often available as over the counter (OTC) in many regions. Due to various strengths of each marketed product, the therapeutic level for DPN management is concerned in terms of efficacy and safety. Currently, there is a few guidance of DPN management at primary care. Therefore, this expert guideline was developed to recommend the primary screening method for early screening and updated scientific data of neurotropic B vitamins in treatment. The ultimate goal of this guideline is to improve DPN patients care to avoid devasting severe complication from delayed treatment.

Certain materials may include the subjective opinions and clinical experiences of individuals. These are not mandatory to adhere to, and healthcare professionals can decide if they are suitable for their institution’s setting in order to provide the best possible outcome for their patients.

## 2. Methods

### 2.1. Study design and expert panel

A working group consisted of an endocrinologist, a neurologist, and three academic pharmacists. Members of the expert panel were selected based on the following criteria: a practicing neurologist or endocrinologist with more than 15 years of experience or pharmacist with the experience in pharmaceutical care; considerable experience in the diagnosis, evaluation, and management of DPN; previous experience as a committee for guideline development. The experts conducted the literature review by searching published data sources, including MEDLINE, the Cochrane Library, CINAHL, PubMed, SCOPUS, and Google Scholar, plus Google and hand-searching. The full text of original research studies related international guidelines, and clinical evidence of the combination of neurotropic B vitamins in DPN treatment were included. Afterward, abstracts and full texts were preliminarily screened with the exclusion criteria of irrelevance, redundancy, and obsolescence. Finally, the included publications were reviewed and discussed in the meeting to establish the consensus statement of ‘DPN management with a combination of neurotropic B vitamins for pharmacists.’

### 2.2. Development and grading of the statements

The expert consensus was structured to cover all aspects of DPN management including goal of treatment, disease screening, treatment, and referral criteria. Data were discussed and summarised into individual recommendation statements on which the experts designated their percentage of agreement from 1 to 10, with 1 representing ‘strongly disagree’ and 10 representing ‘strongly agree’, respectively. Each statement was endorsed if more than 80% of the experts rated the level of agreement at 7 or above (Ziegler et al., 2022). In cases where 100% agreement was not attainable, the group would deliberate on such statements to present additional perspectives or further discussion. This approach served to recognise the complexity of the issue and provide a more nuanced understanding of the available evidence.

## 3. Results

The expert consensus was discussed and agreed into five main statements covering goal of treatment, initial evaluation, referring criteria, and management with a combination of neurotropic B vitamins. The final consensus statements and percentage of agreement were shown in Table 1.

**Table 1.** Expert consensus recommendations for community pharmacists in peripheral neuropathy management.

No	Consensus statement	% Agreement
1	The goals of DPN treatment are (1) to reduce DPN symptoms and its impact on QoL, (2) to prevent or control DPN complications, (3) to minimise adverse reactions from DPN treatments, and (4) to promote patients’ understanding and adherence to the treatment plan.	100
2	The initial evaluation of DPN can be done by	100
2.1	Gathering medical histories, such as duration of symptoms, alcohol consumption, underlying diseases especially diabetes, hypertension, hypothyroidism/hyperthyroidism, cardiovascular diseases, dyslipidemia, chronic kidney disease, obesity, and duration of diabetes.	
2.2	Inquiring about concomitant medications (such as metformin, antacids and proton pump inhibitors), food supplements (such as vitamin B) and the patient’s dietary patterns.	83.3
2.3	Querying about symptoms of DPN including lancinating, paresthaesia, tingling, numbness, electric shock-like sensations, burning, cold allodynia, itching and timing of the symptoms. Such symptoms may occur on both sides of the body (symmetrical)	100
2.4	The Douleur Neuropathique 4 Questions (DN4) interview or the 7-item version of the DN4 questionnaire with a cut-off of 3 may be used for screening painful DPN. If the DN4 score is less than 3, advise patients to see a physician in case of severe pain (NRS = 7–10) or if it impacts their QoL.	83.3
2.5	A simple screening tool for loss of protective sensation (LOPS) is the monofilament test.	100
2.6	If patients have symptoms including weakness, foot drop, hand and/or foot atrophy, and unilateral numbness, they should be advised to consult a doctor to determine more possible causes.	100
3	If DPN is suspected after evaluation regarding consensus statement 2 such as duration of numbness of more than three months with underlying diseases and/or current use of the drug in 2.1 and 2.2, the total symptom score of DN4 is three or more points, and inability to express tactile perception in the monofilament test. A combination of neurotropic B vitamins can be used in therapeutic doses (therapeutic doses of vitamin B1 100–600 mg/day, vitamin B6 100–600 mg/day, vitamin B12 400–5000 mcg/day) by	100
3.1	Monotherapy of a combination of neurotropic B vitamins or	83.3
3.2	In combination with other drugs, such as SNRI, TCA, topical treatment, and alpha-2 delta subunit calcium channel blocker antiseizure medications.	83.3
4	Combined neurotropic B vitamins have synergistic effect and improve patient compliance.	83.3
5	Advise patients on pharmacological and non-pharmacological approaches and take medication regularly.	100
5.1	Patients are advised to self-assess their symptoms after the first 2 weeks of taking a combination of neurotropic B vitamins. If the symptoms do not improve or patients experience intolerant side effects, it is advised to consult a physician for further investigation.	83.3
5.2	Minimise the risk of neurotropic B vitamin deficiency, including avoidance of foods that interfere with the absorption of B vitamins and regular screening for B12 deficiency and supplementation of B12 among strict vegetarians.	83.3

## 4. Discussion

**Consensus statement 1:** The goals of DPN treatment are (1) to reduce DPN symptoms and its impacts on quality of life (QoL), (2) to prevent or control DPN complications, (3) to minimise adverse reactions from DPN treatments, and (4) to promote the patient’s understanding and adherence to the treatment plan.

Worldwide, 25–50% of people with diabetes experience symptomatic DPN (Ziegler et al., 2014). This leads to DPN complications including foot ulcers and amputation contribute to the 73% mortality rate (Malik et al., 2020). According to a recent study, 73.1% of patients with neuropathic pain reported lower quality of life (QoL) on a physical and mental level such as physical discomfort, worsened physical conditions, anxiety, and depression (Jing et al., 2018).

Although the mechanism of DPN remains unclear, certain pathological pathways related to DPN have been hypothesised due to hyperglycaemia, including (1) polyol, (2) hexosamine, (3) protein kinase C, and (4) advanced glycation end-products (AGEs) (Feldman et al., 2017). Those pathways cause inflammatory signalling, mitochondrial dysfunction, and the decline of insulin signalling, which are the three main factors of neuronal apoptosis. The disease also results from intense treatment, known as treatment-induced neuropathy of diabetes (TIND) or insulin neuritis. Hypoglycaemia is the main cause stimulating inflammatory cytokines; therefore, glycemic control should be adequately maintained. Pathologically, injured nerves cause nerve function impairment, resulting in negative symptoms of DPN as well as positive symptoms that increase pain signals from adjacent neurons activated by the upregulation of sodium and calcium channels to compensate for the damaged fibres. The injured nerves can be regenerated, and their functions are restored through several pathways (Balakrishnan et al., 2021; Önger et al., 2016). However, an earlier study demonstrated that these neuron lesions repeatedly occur in people with diabetes who have long-term hyperglycaemic conditions (Boucek, 2006). Moreover, the loss of regeneration activity is recognised as a point of no return, in cases of more than 50% neuronal damage (Nix, 2017). Therefore, the traditional management plan is to prevent or slower the disease progression by optimal glycemic control at an early stage (Boucek, 2006).

The ultimate goals of DPN therapy are the accomplishment of a well-controlled metabolic condition and the alleviation of all painful disorders (Nix, 2017). Standard medications comprise tricyclic antidepressants (TCAs), serotonin and norepinephrine reuptake inhibitors (SNRIs), and antiseizure medications (gabapentin and pregabalin). However, these medications still have concerns in clinical practice. Firstly, these remedies are used for predominantly positive symptoms. Secondly, the safety profiles of TCAs are of particular concern due to their anticholinergic effects, which have the potential for cardiotoxicity and other common side effects such as dry mouth, orthostatic hypotension, constipation, and urinary retention. While SNRIs and calcium channel blocker antiseizure medications are associated with less severe adverse effects like nausea, dizziness, and drowsiness. These limitations can be resolved by using complementary nutraceuticals or dietary supplements such as neurotropic B vitamins, which are essential to manage the symptomatic DPN and minimise adverse reactions from the regimen. Additionally, the combination treatment is also taken to treat overtly negative symptoms (Ang et al., 2018).

**Consensus statement 2:** The initial evaluation of DPN can be done by:

2.1 Gathering medical histories, such as duration of symptoms, alcohol consumption, underlying diseases, especially diabetes, hypertension, hypothyroidism/hyperthyroidism, cardiovascular diseases, dyslipidemia, chronic kidney disease, obesity, and duration of diabetes.
2.2 Inquiring about concomitant medications (such as metformin, antacids and proton pump inhibitors), food supplements (such as vitamin B), and patient’s dietary patterns.
2.3 Querying about symptoms of DPN, including lancinating, paresthaesia, tingling, numbness, electrical shock-like sensations, burning, cold allodynia, itching, and timing of the symptoms. Such symptoms may occur on both sides of the body (symmetrically).
2.4 The Douleur Neuropathique 4 Questions (DN4) interview or the 7-item version of the DN4 questionnaire with a cut-off of 3 may be used for screening painful DPN. If the DN4 score is less than 3, advise patients to see a physician in case of severe pain (Numerical Rating Scale (NRS) = 7–10) or if it impacts their QoL.
2.5 A simple screening tool for loss of protective sensation (LOPS) is the monofilament test.
2.6 If patients have symptoms including weakness, foot drop, hand and/or foot atrophy, and unilateral numbness, they should be advised to see a doctor to find out more possible causes.

### Disease-related DPN

The main risk factors related to DPN are duration of diabetes, hyperglycaemia, age, other underlying diseases and some drugs leading to increased severity (Table 2) (Jones et al., 2019; Papanas & Ziegler, 2015; Pfannkuche et al., 2020). These should be inquired when consulting patients to set the treatment plan.

**Table 2.** Common causes of peripheral neuropathy (Jones et al., 2019; Papanas & Ziegler, 2015).

Patient’s background: hereditary and previous trauma
Toxin: alcohol and cigarette
Disease: Hematological disease Cardiovascular disease; vasculitis, hypertension, and dyslipidemia Metabolic derangement; overweight, obesity, hyperglycaemia, hyperthyroidism/ hypothyroidism Infections; HIV, diphtheria, and hepatitis B Autoimmune disease Chronic inflammation Malignancy Kidney disease
Drug: Cardiovascular agents; statins and amiodarone Chemotherapeutic agents; platinum compounds, vinca alkaloids, taxanes Antibiotics; isoniazid, ethambutol, linezolid, metronidazole, interferons, leflunomide Anti-HIV (NRTIs); zalcitabine, didanosine, stavudine, lamivudine Drug-induced vitamin B12 deficiency; proton-pump inhibitors, H2-receptor antagonists and antacids

Abbreviation: human immunodeficiency virus (HIV), nucleoside/nucleotide reverse transcriptase inhibitors (NRTIs).

### Drug-related DPN

Vitamin B deficiency due to diabetes mellitus and its treatment can result in DPN. For instance, metformin was significantly associated with vitamin B12 deficiency (adjusted OR [95% CI]: 2.92 [1.26–6.78]) (Reinstatler et al., 2012). Therefore, the American Diabetes Association (ADA) recommended the preventive administration of vitamin B12 for long-term use of metformin due to the tendency for DPN incidence (American Diabetes Association, 2023b). Additionally, this deficiency can be induced by other medications such as proton-pump inhibitors, H_2_-receptor antagonists, and antacids (Table 2) by suppressing B12 absorption (Miller, 2018).

Although vitamin B is water-soluble, misuse of vitamins may result in accumulation and toxicity. Hypervitaminosis of B vitamins probably exhibits skin hypersensitivity, headache, gastrointestinal disturbance, and hyperuricaemia (Saljoughian, 2021). Therefore, it is crucial to ask the current vitamin supplements being used to increase patient safety and prevent overdose toxicity.

### DPN screening

The earlier and faster DPN is recognised and diagnosed, the more treatment options are available. The ADA and the Canadian Diabetes Association's guidelines both recommend various types of DM-based screening intervals for DPN. People with type-1 diabetes require DPN evaluation after 5 years of the diagnosis and then every year, while type-2 diabetes must be assessed at diagnosis and annually (American Diabetes Association, 2023a; Diabetes Canada Clinical Practice Guidelines Expert Committee, 2018). Patient history, neuropathic signs/symptoms, and preliminary tests are simply employed as a DPN screening tool to review neuronal function and protective sensation of both small and large fibres (Pop-Busui et al., 2017). Up to half of the patients may experience symptoms of DPN, while the rest are asymptomatic. Sometimes, only diagnostic investigation may reveal that they are facing certain positive neuropathic symptoms such as burning discomfort, altered temperature sensation, hyperaesthesia, tingling, prickling, sudden shooting and stabbing sensations (Diabetes Canada Clinical Practice Guidelines Expert Committee, 2018; Yavuz, 2022).

At an early stage, neuropathies gradually develop and insidious injury to the distal sensory nerves, followed by peripheral sensory loss involving more proximal extremities. Eventually, motor nerves will be disturbed when diabetes is insufficiently controlled (Zochodne et al., 2008). This process results in loss of protective sensation (LOPS), such as minor physical, thermal, or chemical injuries (Tunis et al., 2001). Generally, 10-g monofilament is used to test LOPS, by touching the soles of patients’ feet with the device while closing the eyes, followed by reporting their tactile sensation. If the patient is unable to express touch perception, they could have negative symptoms of DPN (American Diabetes Association, 2023a). The use of 10-g monofilament by community pharmacists should be considered based on the country regulation in terms of patient care.

A screening process by community pharmacy can improve patient treatment and referral to a general practitioner or specialist, if necessary. There are screening questionnaires used in pharmacies for detecting early stage of DPN, such as, the United Kingdom screening test (UKST) and the Norfolk Quality of Life Questionnaire-DPN (Norfolk QOL-DN).

**Figure 1.**
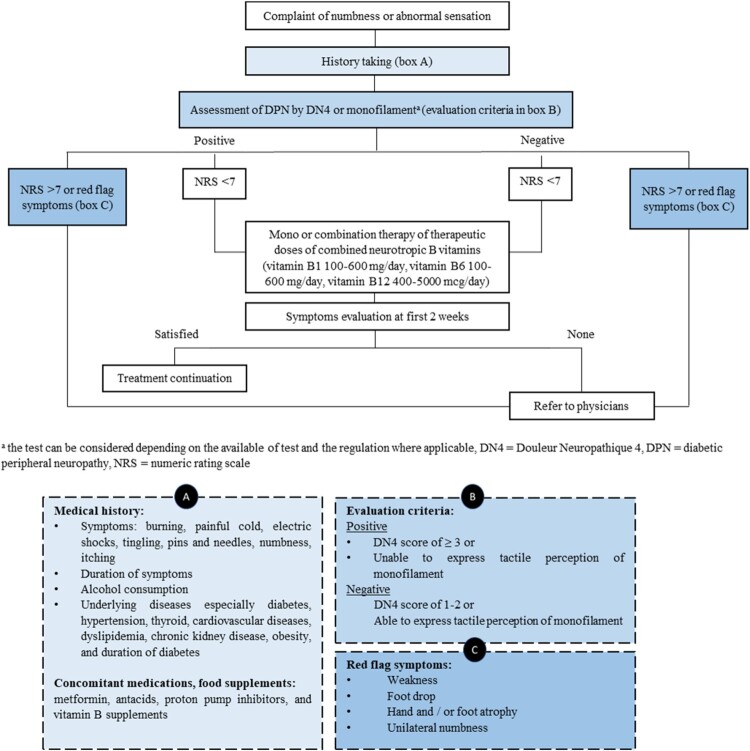
Overview of the preliminary evaluation and management of DPN.

The DN4 questionnaire is an alternative tool for screening painful DPN. It consists of four questions with ten items of DPN. This tool is appropriate for community pharmacies since it is simple but has acceptable reliability (inter-rater agreement coefficient: 0.80, and test-retest intra-class correlation coefficient: 0.95) and high specificity (90%) (Perez et al., 2007). The first seven interview items include both positive and negative symptoms, such as burning, electrical shock-like, cold allodynia, tingling, numbness, or itching. The other three items are the neurological examination of the pain area such as touch hypoesthesia, pinprick hypoesthesia, and tactile allodynia (Bouhassira et al., 2016). In context of a community pharmacy setting, the DN4 interview using only a 7-item format for patient interview without the clinical examination part could be applied for preliminary diagnosis (Figure 1). The cut-off value of 3 or more points defines neuropathic pain with a sensitivity of 78.0% and a specificity of 81.2% (Bouhassira et al., 2005). Coupled with the DN4 questionnaire, patients were typically requested to report their pain experienced in the past seven days using a reliable 11-point NRS. This scale ranges from 0, indicating no pain, to 10, which represents the most severe pain imaginable (1–3 mild pain, 4–6 moderate pain, 7–10 severe pain) (Bouhassira et al., 2005).

The certain severe cases, particularly those involving severe pain (NRS = 7–10) or symptoms that negatively impact an individual's QoL, might be beyond the scope of community pharmacists, for example, ‘foot drop’, which is a weak or paralysed condition of muscles responsible for lifting the front part of a foot. This may suggest the superficial peroneal nerve damage (Westhout et al., 2007). Additionally, the presence of muscle atrophy, painful cramps, and unilateral numbness strongly indicates the involvement of affected lower motor neurons (Scott & Kothari, 2005). In this circumstance, a pharmacist must refer the patient to physician for further investigation.

**Consensus statement 3:** If DPN is suspected after evaluation regarding consensus statement 2, such as a duration of numbness of more than three months with underlying diseases and/or current use of the drug in 2.1 and 2.2, the total symptom score of DN4 is three or more points, unable to express tactile perception in the monofilament test. A combination of neurotropic B vitamins can be used in therapeutic doses (proposed experimental dose-ranging of vitamin B1 100–600 mg/day, vitamin B6 100–600 mg/day, vitamin B12 400–5000 mcg/day) by

3.1. Monotherapy of a combination of neurotropic B vitamins or
3.2. In combination with other drugs such as SNRIs, TCAs, topical treatment, and alpha-2 delta subunit calcium channel blocker antiseizure medications.

In general, the management of DPN includes three cornerstones: (1) causal treatment, including lifestyle modification, intensive diabetes therapy, and multifactorial cardiovascular risk intervention, (2) pathogenetically oriented pharmacotherapy using specific nutraceuticals, and (3) symptomatic treatment of neuropathic pain (Pop-Busui et al., 2022).

The first-line treatment options for symptomatic neuropathic pain comprise antidepressants, including TCAs (amitriptyline, nortriptyline, and imipramine) and SNRIs (duloxetine), as well as calcium channel blocker antiseizure medications (pregabalin and gabapentin) (Fornasari, 2017). The combination of neurotropic B vitamins can be used as an alternative choice or adjunctive treatment with standard therapy to help reduce nerve pain and supplemental vitamin deficiency (Abdelrahman & Hackshaw, 2021). Generally, vitamins B1 (thiamine), B6 (pyridoxine), and B12 (cobalamin) have exhibited their neuro-regenerative effects. Therefore, these vitamins are commonly referred to as ‘neurotropic’ B-vitamins due to their affinity for and ability to nourish and support nerve cells (Baltrusch, 2021). Vitamin products can be classified as food supplement and drug where the county specific regulation is applied (examples in table 3). Therefore, it is important to check the vitamin content for the purpose. The recommended daily allowance (RDA) for food supplement purpose depends on gender, age, size, and activity in a person. For example, RDA of vitamin B1, B6 and B12 are 0.3–1.4 mg/day, 0.3–2.0 mg and 0.5–2.8 mcg, respectively (Bureau of Nutrition, 2020; US Food and Drug Administration, 2018). On the other hand, for DPN management, the use of a combination of neurotropic B vitamins should be considered based on published clinical research to achieve a therapeutic dose.

**Table 3.** An example of vitamin B content in the marketed products.

Vitamin	Brand A	Brand B	Brand C	Brand D	Brand E	Brand F
B1 (Thiamine)	100 mg	5 mg	100 mg	50 mg	7.5 mg	7.5 mg
B2 (Riboflavin)		2 mg		50 mg	8.5 mg	8.5 mg
B3 (Niacin)		20 mg		50 mg	60 mg	60 mg
B5 (Pantothenic acid)				45.8 mg	20 mg	20 mg
B6 (Pyridoxine)	7.5 mg	2 mg	200 mg	50 mg	10 mg	10 mg
B7 (Biotin)				50 mcg	50 mcg	50 mcg
B9 (Folic acid)				100 mcg	200 mcg	200 mcg
B12 (Cobalamin)	75 mcg		200 mcg	50 mcg	10 mcg	10 mcg
Other components				Inositol, choline	Inositol, choline	Vitamin C

Several studies of various combination of neurotropic B vitamins have been conducted. For example, a meta-analysis of 226 clinical trials indicated that B vitamin combinations positively affect neurophysiological manifestations and/or activities in people with diabetes (Liew et al., 2019). Tong (1980) documented that the nerve conduction velocity was influenced by a combination of neurotropic B vitamins in a dose-dependent manner (Tong, 1980). A prospective non-interventional study in Indonesia revealed that a once daily fixed-dose combination of B vitamins (100 mg vitamin B1, 100 mg vitamin B6, and 5 mg vitamin B12) showed a significant reduction in Total Symptom Score (TSS) (*p* < 0.001) of numbness, stabbing pain, burning pain, and paresthaesia within 14 days (Hakim et al., 2018). Previous studies showed that neuropathic B vitamins (100 mg of vitamin B1, 200 mg of vitamin B6 and 200 mcg of vitamin B12) either two or three times a day significantly improved test of the perception of temperature sensation and improved painful symptoms with more than 87% responded patients respectively (Janka et al., 1991; Rizvi et al., 2013).

**Consensus Statement 4:** Combined neurotropic B vitamins have a synergistic effect and improve patient compliance.

Each neurotropic B vitamin has the specific role in nerve growth and regeneration. Vitamin B1 (thiamine) is one of the cofactors in glucose metabolism, which provides adequate energy to neurons by non-pathogenic pathway (pentose phosphate pathway). the deficiency status by alcoholic beverages, diuretic agents, and dialysis for kidney disease affects almost all body systems, including the metabolic, neurologic, cardiovascular, respiratory, gastrointestinal, and musculoskeletal systems (Smith et al., 2021; Suter & Vetter, 2000).

Vitamin B6 (pyridoxine) relates to neurotransmitter synthesis. The form of pyridoxal 5′-phosphate is the most important coenzyme variance, associated with GABA, dopamine, and serotonin synthesis (Baltrusch, 2021; Calderón-Ospina & Nava-Mesa, 2020). In addition, pyridoxine has an analgesic effect due to its participation in the presynaptic inhibition of neurotransmitter release from the nociceptive afferent fibres. Therefore, a pyridoxine deficiency mainly causes several complications in the central nervous system, including seizures, depression, and mental status changes (Calderón-Ospina & Nava-Mesa, 2020).

Vitamin B12 (cobalamin) plays an essential role in the synthesis and maintenance of myelin sheaths as well as neuron recovery. Many forms of vitamin B12 exist such as cyano-cobalamin, methyl-cobalamin, adenosyl-cobalamin, or hydroxycobalamin which are metabolised to cobalamin as a coenzyme in these processes. A systematic review and meta-analysis indicated that low levels of vitamin B12 were significantly associated with neuropathy events (OR [95% CI]: 1.51 [1.23–1.84]) (Stein et al., 2021). Although excessive cobalamin is accumulated in the liver which reduces the likelihood of depletion, malabsorption of vitamin B12 can cause certain haematologic and neurological complications (Wolffenbuttel et al., 2019).

While vitamin B1 acts as a site-directed antioxidant, vitamin B6 balances nerve metabolism, and vitamin B12 maintains myelin sheaths (Calderón-Ospina & Nava-Mesa, 2020), using a combination of neurotropic B vitamins is more advantageous than individual vitamin products due to synergistic effect and improved patient compliance (Calderón-Ospina & Nava-Mesa, 2020).

The supportive *in vitro* study showed that a combination of high-dose neurotropic B vitamins enhanced the neuronal outgrowth of murine dorsal root ganglia much stronger than the individual supplement (Fujii et al., 1996). In animal study, it reported that sole vitamin B12 or in combination with B1 and B6 has a more powerful neuron regenerative effect than vitamin B1 or B6 alone on damaged peripheral nerves (Al-Saaeed et al., 2019). A combination of neurotropic B vitamins tends to have lower toxicity than a high dose of individual vitamin B. Since a combined product is a fixed-dose tablet containing approximately 100 mg of vitamin B1, 200 mg of vitamin B6, and 200 mcg of vitamin B12, an earlier safety study reported that taking a megadose of vitamin B monotherapy may result in several adverse reactions (Calderon-Ospina et al., 2020). This aspect is discussed in detail in a subsequent section.

**Consensus statement 5:** Advise patients on pharmacological and non-pharmacological approaches and take medication regularly.

5.1. Patients are advised to self-assess their symptoms after the first 2 weeks of taking a combination of neurotropic B vitamins. If the symptoms do not improve or patients experience intolerant side effects, it is advised to consult a physician for further investigation.
5.2. Minimise the risk of neurotropic B vitamin deficiency, including avoidance of foods that interfere with absorption of B vitamins and regular screening for B12 deficiency and supplementation of B12 among strict vegetarians.

Most clinical evidence and real practice showed improved symptom scores within 1–2 weeks after a combination of neurotropic B vitamin administration (Hakim et al., 2018; Janka et al., 1991; Kuhlwein et al., 1990; Vetter et al., 1988). The duration of DPN treatment with a combination of neurotropic B vitamins depends on the underlying cause and severity of the symptoms. A combination of neurotropic B vitamins (B1, B6, and B12) is well established for DPN management and safe for long-term use (Hakim et al., 2018). This could imply that the course of a combination of neurotropic B vitamins can be continued if it is effective as an initial remedy for DPN symptoms and re-assessment of symptoms. The safety profile of each vitamin B was discussed as followed.

There have not been any adverse effect reports on oral vitamin B1. It hypothesised that gastrointestinal absorption of an excessive dose of thiamine could be saturated (more than 5 mg) (Calderon-Ospina et al., 2020).

Regarding pyridoxine, a very high dose (>500 mg/day) and/or prolonged treatment (>6 months) has been clinically reported with neurotoxic side effects but is rare and reversible after discontinuation (Calderon-Ospina et al., 2020; Echaniz-Laguna et al., 2018). There were only three Thai patients aged 80, 83, and 83 years old who used 600 mg/day of vitamin B 6 for 3–10 years, experienced sensory neuropathy and axonal sensorimotor polyneuropathy, leading to sensory ataxia. Likewise, a series of clinical reports revealed that long-term and large doses (>2 g daily for many months to years) correspond to dose-dependent pyridoxine-induced neuropathy (Kulkantrakorn, 2014). Several proposed mechanisms are related to an increase in the metabolite of pyridoxine (quinone methide-type intermediates) and a reduction in the biosynthesis of GABA neurotransmission due to pyridoxal kinase mutations (Hadtstein & Vrolijk, 2021). Although few case reports have addressed the correlation between vitamin B6/B12 intake and adverse complications (hip fracture and lung cancer risk), there is insufficient evidence to conclude these harmful occurrences (Calderon-Ospina et al., 2020).

Apart from pharmacological treatment, community pharmacists should provide counselling on nutrition, lifestyle modification, footcare and non-pharmacotherapy. There is strong evidence suggesting that a high-fat diet can cause signs of neuropathy in mice (Obrosova et al., 2007; Rumora et al., 2022). Some foods tend to interact with the absorption and metabolism of B vitamins, resulting in decreased B vitamin levels. For example, shellfish, clams, some freshwater fish viscera, crustacea, and certain ferns contain thiaminases which hydrolyses thiamine (Thurnham, 2005). Additionally, coffee, tea, and energy drinks contain caffeic acid and tannic acid can cause impairing thiamine absorption (Marrs & Lonsdale, 2021). When vitamin B is consumed in diets that contain high levels of calcium or fibre, the absorption process is disrupted because of the complex formation (Ioniță-Mîndrican et al., 2022). Cobalamin deficiency has also been reported due to insufficient vitamin B12 from plant-based sources in vegetarian and vegan populations, although the daily requirement for healthy vegetarians is 2.4 mcg of cobalamin. To follow the recommendation, a vitamin B12 tablet dose of 50–100 mcg daily or 2000 mcg weekly should be supplemented (Rizzo et al., 2016).

Lifestyle modifications, such as regular exercise, a balanced diet, cessation of smoking and alcohol consumption, are recommended in DPN patients to prevent DPN progression and other complications (Smith et al., 2022). Low-impact exercise programmes such as walking, swimming, and cycling, significantly benefit all DPN stages, such as DPN risk reduction in people with prediabetes, improvement of affected nerves, and symptom relief in patients with DPN (Balducci et al., 2006; Streckmann et al., 2022). Moreover, lifestyle modifications for reduce risk of hypoglycaemic event are also important for DPN patients such as eating food on time, avoiding fasting for each meal, eating small but frequent meals, increasing food intake before intensive exercise and counselling for patient to recognised the signs and symptoms of hypoglycaemia (Evans Kreider et al., 2017). Regularly checking patients’ feet for cuts, sores, or swelling, and wearing proper footwear to prevent foot ulceration. Appropriate footwear should fit, protect and accommodate the shape of foot. Moreover, diabetes patients should wear socks within footwear to reduce shear and friction (van Netten et al., 2018). Educational sessions and counselling are the major role of community pharmacists to enhance patient compliance with both pharmacological and non-pharmacological programmes. Together, these comprehensive interventions have many advantages for disease-related conditions, resulting in a significant decrease in the severity of DPN when compared with a control group that did not receive the intervention (*p* < 0.001) (Ghavami et al., 2018). Non-pharmacological treatment that may be educated DPN patients to consults physician in hospital for adjunctive treatment with pharmacotherapy such as spinal cord stimulation or short-term frequency-modulated electrical stimulation (Amato Nesbit et al., 2019).

In essence, community pharmacists offer a range of services, from medication management to lifestyle counselling and education. While the outcomes for diabetic care in community or primary care settings were more favourable, there is currently a lack of emphasis on managing microvascular complications. There appears to be strong support for implementing a novel screening, monitoring, and management service (Woodhams et al., 2023).

This guideline was adopted as an opportunity to offer expanded and challenging services in developing countries. Even in countries with fewer issues, it may be necessary to verify the effective dosage. Nevertheless, there is currently a lack of studies on cost-effectiveness, and additional data may be necessary.

## 4. Conclusion

The increasing prevalence of diabetes and its complications, such as diabetic polyneuropathy (DPN), poses significant public health challenges worldwide. Despite advances in the understanding of the DPN, the condition is still underdiagnosed and inadequately treated. Therefore, it is essential to implement effective strategies for early detection and prevention of DPN to reduce its burden and associated sequelae. This article offers recommendations for the screening, diagnosis, and treatment of DPN by pharmacists in primary care services.
